# Hydropinotherapy with Sulphurous Mineral Water as Complementary Treatment to Improve Glucose Metabolism, Oxidative Status, and Quality of Life

**DOI:** 10.3390/antiox10111773

**Published:** 2021-11-05

**Authors:** Maria Costantino, Valeria Conti, Graziamaria Corbi, Amelia Filippelli

**Affiliations:** 1 Department of Medicine, Surgery and Dentistry ‘Scuola Medica Salernitana’, University of Salerno, Via S. Allende, 84081 Baronissi, Italy; vconti@unisa.it (V.C.); afilippelli@unisa.it (A.F.); 2 Association Non-Profit F.I.R.S.Thermae (Interdisciplinary Training, Researches and Spa Sciences) in Italian National Register of Research of MIUR, 80078 Pozzuoli, Italy; 3 Department of Medicine and Health Sciences, University of Molise, 86100 Campobasso, Italy; graziamaria.corbi@unimol.it

**Keywords:** sulphurous mineral water, glycaemia, oxidative stress, ROMs, quality of life

## Abstract

Hydropinotherapy is a salus per aquam (Spa) treatment suitable as a complementary approach to treat several diseases, which strongly affect the quality of life (QoL). Hydropinotherapy with sulphurous mineral water exerts benefits thanks to components, such as hydrogen sulphide, which is considered mainly responsible for antioxidant and hypoglycaemic effects. Such properties, linked from each other, could favour an improvement in patients’ QoL. However, data on humans are scarce. This study aimed to investigate whether a cycle of sulphurous hydropinotherapy was able to modify plasma levels of glucose and reactive oxygen metabolites (ROMs) and improve QoL in patients suffering from several chronic disorders. A prospective, observational study involved patients with gastrointestinal diseases who received a prescription of a cycle of sulphurous hydropinotherapy (S-HT). Age- and sex-matched control group was enrolled (No S-HT). Glycaemia and plasma concentration of ROMs were measured in all subjects. The impact of spa treatment on the QoL was assessed using the Short Form 36 Health Status Survey questionnaire (SF-36). All parameters were measured at baseline and at the end of a 2-week treatment. Between the groups, no differences were found in glycaemia and ROMs at baseline. In the S-HT group, a reduction in glycaemia and ROMs, both in respect to baseline (*p* = 0.005 and *p* = 0.031, respectively) and to control group, as shown by the delta value calculated, as the difference between the values at 2 weeks and baseline (*p* = 0.0009 and *p* = 0.0001, respectively). In the S-HT, delta ROMs was the best predictor of delta glycaemia with a direct linear correlation (beta = 0.559, 95% CI 0.471 to 0.647, *p* < 0.0001). In the S-HT, the SF-36 total score was improved both when compared with baseline (*p* = 0.002) and with No S-HT (*p* = 0.001). Sulphurous hydropinotherapy induces a decrease in glycaemia and ROM levels, also ameliorating the patients’ QoL. Therefore, it could be considered a useful complementary therapeutic approach.

## 1. Introduction

Hydropinotherapy is a salus per aquam (Spa) treatment consisting of drinking quantities of mineral water at a definite temperature, according to specific prescriptions [1,2,3]. 

The most used waters are the sulphurous mineral waters which, in addition to chemical components such as bicarbonate, calcium and magnesium, contain a quantity of hydrogen sulphide (H_2_S) higher than or equal to 1 mg per litre [1,2]. Several studies have highlighted that sulphurous mineral waters increase hepatic glycogen and reduce blood glucose, probably owing to a sulphur-induced vagal stimulation with a consequent increase in insulin secretion [2,4].

Moreover, both in animal models and humans, it has been demonstrated that sulphurous mineral water exerts an antioxidant action by reducing the oxidation of biomolecules with a consequent improvement of the cellular redox state [5,6,7,8,9,10,11,12,13]. This can favour protection against the imbalance between oxidants and antioxidants molecules, referred to as oxidative stress, which is one of the most important hallmarks of ageing and chronic diseases [14,15]. 

Impairment in glucose homeostasis, especially over a long period, and oxidative stress, may activate metabolic pathways with a pathogenetic role in the onset and progression of type 2 diabetes (T2D) [4,16]. Therefore, interventions aiming to regulate glucose metabolism and decrease oxidants amount represent an opportunity to delay or contrast such risks [17].

Sulphurous mineral water exerts several beneficial effects, including antioxidant and hypoglycaemic ones [18,19,20,21].

It is important to underline that T2D, as well as other chronic diseases, harms the quality of life (QoL), especially in presence of comorbidity [22,23]. 

Contrasting oxidative stress accumulation, as well as hyperglycaemia, may be helpful to improve the patients’ quality of life (QoL) [24,25].

The World Health Organisation (WHO) defines QoL as ‘individuals’ perception of their position in life in the context of the culture and value systems in which they live, and in relation to their goals, expectations, standards and concerns’ [26].

However, several definitions of QoL have been proposed with the effort to consider the variety of aspects encompassing both positive and negative lived experiences [27]. 

Given such complexity, there is a need to use methods and instruments able to provide a multi-dimensional assessment, such as the Short Form 36 Health Status Survey (SF-36). The SF-36 is the most used questionnaire to evaluate the QoL. It is characterised by shortness and precision and is a valid and reproducible means, validated in Italy by the Mario Negri Institute [28,29,30,31,32].

Several interventions have been recognised to be helpful in ameliorating QoL [33], including those leading to improvements of oxidative stress and glucotoxicity, such as exercise-based [34] and medical education interventions [24]. 

It has been suggested that Spa treatments favour a significant improvement of the QoL of patients with chronic diseases [21,35,36].

However, contrary to other Spa treatments based on the use of sulphurous mineral water, data regarding the effects of sulphurous hydropinotherapy on glucose and redox homeostasis, as well as on patients’ QoL, are scarce. Therefore, this study aimed to investigate whether a cycle of hydropinotherapy with sulphurous mineral water was able to modify oxidative status and glycaemia and improve QoL in patients who received a prescription of such a Spa treatment.

## 2. Methods

### 2.1. Study Population and Study Design

This is a prospective observational study involving patients with a history of gastrointestinal diseases who received (S-HT group), or not (No S-HT group), a prescription for a cycle of hydropinotherapy with sulphurous mineral water by their physicians, either general practitioners (GPs) or specialists.

The study obtained the approval of the Ethics Committee (No. 7 r.p.s.o./2020) Campania Sud, Naples, Italy, according to the Declaration of Helsinki and its amendments. To collect patients’ data, a specific case report form (CRF) was used.

All recruited subjects fulfilled the following inclusion criteria: age ≥ 18 years; history of chronic gastrointestinal pathologies (i.e., chronic gastritis/gastroduodenitis, gastroesophageal reflux, and hiatal hernia); signed informed consent form.

Exclusion criteria were the presence of acute clinical conditions; cancer and autoimmune diseases.

The patients (S-HT) underwent a 2-week cycle of hydropinotherapy with sulphurous mineral water which includes an appreciable number of bicarbonates, calcium, and magnesium, in addition to bivalent sulphur and its compounds (11.7 mg/L hydrogen sulphide, 1.962 mg/L bicarbonate ions, 505 mg/L calcium ions, 83.4 mg/L magnesium ions; 6.08 pH). A detailed description of the characteristics of the mineral water is reported in Table S1.

According to medical prescription, this Spa treatment consisted of a daily intake, in sips and at room temperature, of 1–3 glasses (with a capacity of 250 mL) of sulphurous mineral water with a rest interval of 10–15 min. The treatment was performed at the Telese Spa (Benevento, Italy). The patients stayed at the spa just long enough to undergo the treatment (about 1 h). 

Patients of the control group (No S-HT), sex- and age-matched individuals suffering from the same clinical conditions did not receive any integration with sulphurous hydropinotherapy of their normal daily hydration and did not stay at the Spa at all.

### 2.2. Measurements

The following parameters were evaluated at baseline and after 2 weeks:

Fasting glycaemia, expressed in mg/dL, using a glucometer.

Plasma concentration of reactive oxygen metabolites (ROMs) using d-ROMs test (Diacron International, Grosseto, Italy). The d-ROMs is a spectrophotometric test used to determine the concentration of ROMs, mainly hydroperoxides (ROOH) [37,38]. ROMs are relatively more stable than reactive oxygen species (ROS) and, therefore, simpler to be detected and quantified [37]. The detailed description of the procedure is described in Costantini and Dell’Omo [39]. Normal plasma ROMs levels, expressed in U.Carr. (1 U.Carr. = 0.08 mg/L of H_2_O_2_), are included in a range of 250–300 U.Carr., while values higher than 300 U.Carr. are considered pathological values [37,38].

The impact of Spa treatment on the quality of life using the Short Form 36 Health Status Survey questionnaire (SF-36). This test consists of a set of measures with 8 items examining different aspects of the self-reported health status. These 8 items explore vitality, physical functioning, bodily pain, general health perceptions, physical role functioning, emotional role functioning, social role functioning, and mental health. Each item is assigned a score ranging from a minimum of 0 (corresponding to poor health) to a maximum of 100 (corresponding to optimal health).

Moreover, the occurrence of undesired events was recorded during the entire study period.

### 2.3. Data Analysis

A descriptive analysis of the general characteristics of the study population was performed. For continuous variables, the results, expressed as mean ± standard deviation (SD), were analysed with Student’s *t*-test for paired and unpaired normally distributed data, and with the Wilcoxon’s signed-rank test for variables with non-normal distribution. The categorical variables were analysed by using the χ^2^ test. Delta values (i.e., difference between levels after 2 weeks and at baseline) were calculated to check the real benefit achieved. Multivariate analyses were performed when adequate. A *p* value < 0.05 was considered statistically significant. Data were analysed using the STATA 16 statistics package.

## 3. Results

All subjects included in the S-HT group were patients who had received a prescription of a cycle of sulphurous hydropinotherapy being affected by gastrointestinal disorders.

The S-HT group included 90 subjects with a mean age of 58 ± 10.9 years (age range: 23–84 years), a BMI = 26.5 ± 3.6, 73% males and 27% females (Table 1). The No S-HT group consisted of 35 subjects, with a mean age of 61 ± 10.7 years (age range: 22–84 years), a BMI = 26.8 ± 4.3, 66% male and 34% female (Table 1). All enrolled subjects were Caucasians.

Between the groups at baseline, no differences were found in glycaemia and ROM levels, as well as in the prevalence of diseases or pharmacological therapy (Table 1). 

### 3.1. Undesired Events

All the subjects of the S-HT group completed the 2-week hydropinotherapy cycle. Only in one subject, an increase in belching was observed on the first day of treatment; however, the subject completed the treatment.

In the subjects enrolled in the No S-HT group, no undesired events were reported. 

### 3.2. Effect of Hydropinotherapy on Glycaemic Levels and Plasma (ROMs)

In 60/90 subjects of the S-HT group and 35 of the No S-HT group, glycaemic levels were assessed at baseline and after 2 weeks. Therefore, the statistical analysis was performed on this subpopulation. Table 2 reports the baseline characteristics of both groups. No differences were found between the groups in glycaemia and ROMs levels at baseline (Table 2), as well as in the prevalence of pathological conditions or pharmacological therapy (Table 2). 

**Figure 1:**
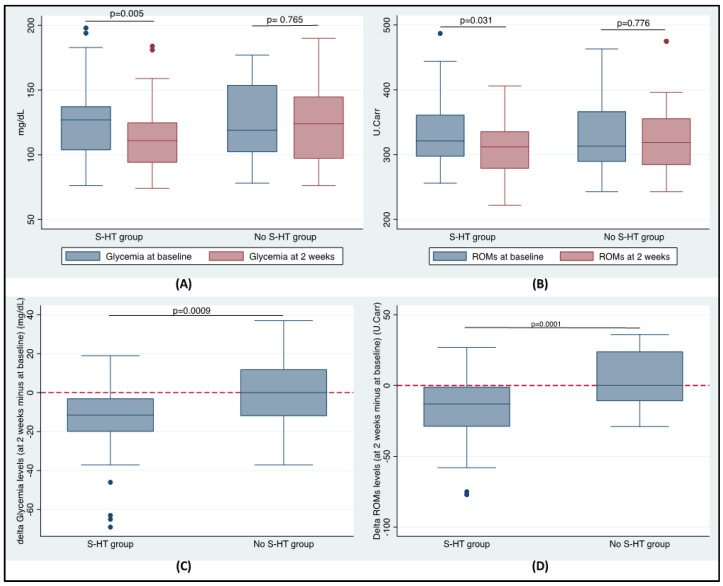
(**A**) Glycaemia and (**B**) ROMs median values at baseline and at 2 weeks in the two groups, and differences in the (**C**) glycaemic and (**D**) ROMs delta value between the groups. Delta value was calculated as the difference between levels at 2 weeks and levels at baseline.

In the S-HT group after 2 weeks, hydropinotherapy was able to significantly reduce the glycaemic values, both in respect to the levels at baseline (*p* = 0.005; Figure 1A) and to those of the No S-HT group, as shown by the delta value calculated as the difference between the values at 2 weeks and baseline (*p* = 0.0009; Figure 1C). Similarly, the treatment was also able to modify the ROMs levels, with a significant reduction both in respect to the levels at baseline (*p* = 0.031; Figure 1B) and to those of the No S-HT group, as shown by the delta value calculated as the difference between the values at 2 weeks and at baseline (S-HT Group: −17.3 ± 28.72 vs. No S-HT Group: 4.66 ± 20.06, *p* = 0.0001; Figure 1D). In the No S-HT group, no changes were found comparing the levels at baseline to the ones at 2 weeks both for glycaemia (*p* = 0.765; Figure 1A) and ROMs (*p* = 0.776; Figure 1B).

**Figure 2:**
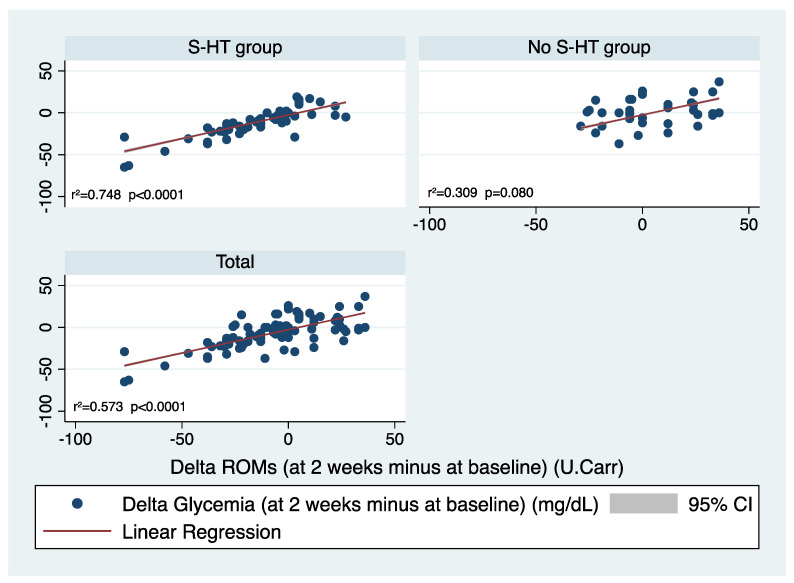
Linear regression results in the S-HT and No S-HT groups, and in the overall population.

Moreover, a multivariate linear regression analysis was performed to assess the possible predictors of the glycaemic level’s improvement, expressed as delta value in the overall population. Therefore, using delta glycaemia as dependent variable and age, sex, BMI, number of drugs, and delta ROMs as independent factors, we found that delta ROM was the best predictor of delta glycaemia, with a direct linear correlation (beta = 0.507, 95% CI 0.414 to 0.601, *p* < 0.0001). Then, to verify if this relationship changed with the treatment, we performed the same analyses stratifying for the groups. We found that in the S-HT group, delta ROM was the best predictor of delta glycaemia with a direct linear correlation (beta = 0.559, 95% CI 0.471 to 0.647, *p* < 0.0001), while in the No S-HT group, the best predictor was the male sex (beta = −14.196, 95% CI −25.452 to −2.939, *p* = 0.015). In Figure 2, the linear regression results are shown in the groups and the overall population. 

Moreover, logistic regression analyses were performed to identify the possible role of sulphurous hydropinotherapy. The results showed that the model fits the data significantly better than a null model (χ^2^(6) = 21.63, *p* = 0.0014). We found that the improvement in ROM levels was a positive and significant (*b* = 0.0369, s.e. = 0.015, *p* = 0.017) predictor of the probability to belong to the S-HT group. For every one-unit increase in delta ROMs, the odds of belonging to S-HT change by a factor of 1.038 (Figure S1).

### 3.3. Impact of Spa Treatment on the Quality of Life (QoL)

**Figure 3:**
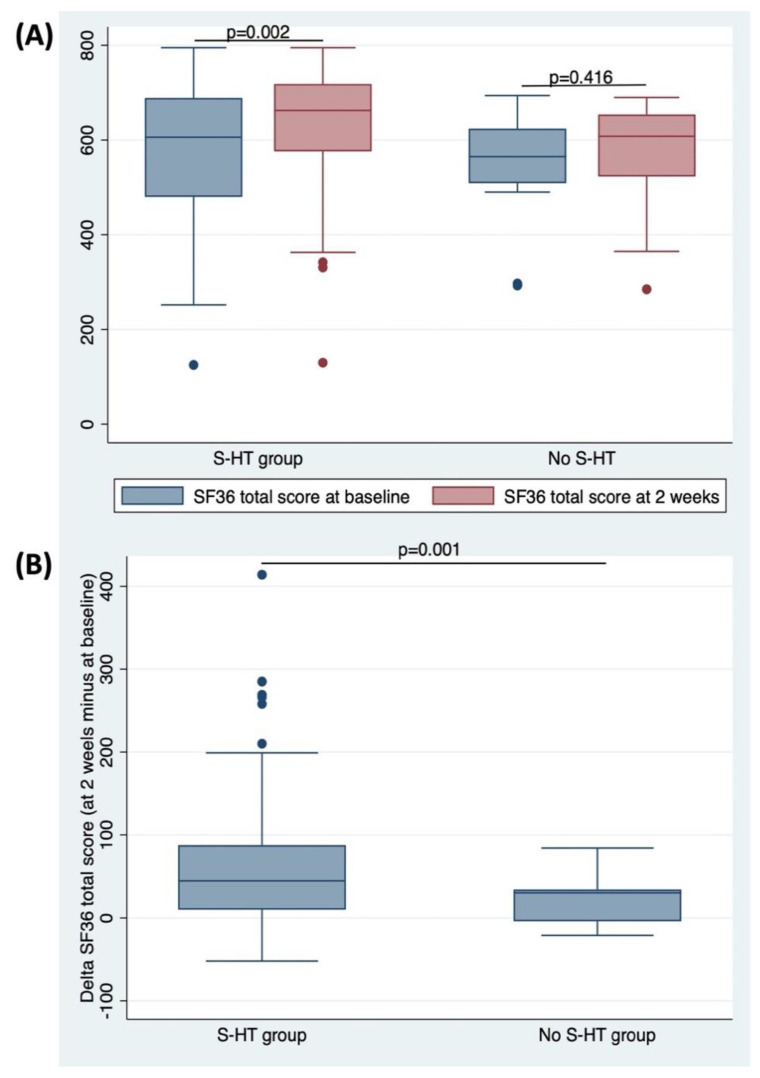
(**A**) SF-36 total score at baseline and at 2 weeks by groups; (**B**) differences in delta SF36 total score between the groups. Delta value was calculated as the difference between scores at 2 weeks and levels at baseline.

By considering the effects of the treatment on QoL, measured using the SF36, in the S-HT group after 2 weeks, sulphurous hydropinotherapy was able to significantly increase the SF-36 total score, both in respect to the levels at baseline (*p* = 0.002; Figure 3A) and to those of the No S-HT, as shown by delta value calculated as the difference between the values at 2 weeks and at baseline (*p* = 0.001; Figure 3B). In the No S-HT group, no changes were found comparing the levels at baseline to the ones at 2 weeks (*p* = 0.416; Figure 3A). The analysis of the single SF-36 items showed that except for ‘physical functioning’ and ‘vitality’, the S-HT group experienced an improvement in every single parameter, while no changes were found in the No S-HT group (Table 3). 

**Figure 4:**
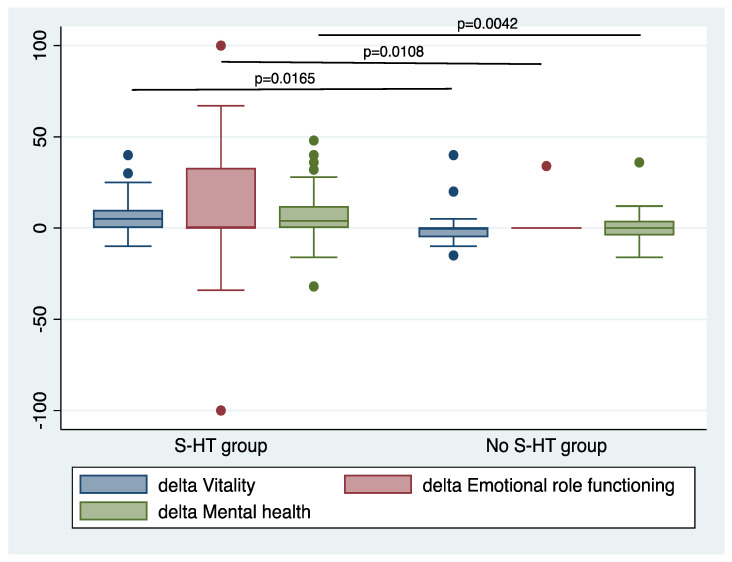
SF-36 Items showing significant differences in delta values between the groups. Delta value was calculated as the difference between scores at 2 weeks and scores at baseline.

To better define the crude effect, the delta value (as a difference between the score at 2 weeks minus the score at baseline) was calculated for each item. The comparison between the groups showed significant differences in the ‘vitality’ (*p* = 0.0165), the ‘emotional role functioning’ (*p* = 0.0108), and the ‘mental health’ (*p* = 0.0042) items, with a greater improvement in the S-HT group in respect to the No S-HT group (Figure 4). 

The reliability and validity of the SF-36 Health Status Survey are reported in the Supplementary Materials (Table S2).

## 4. Discussion

Our study shows that a 2-week sulphurous hydropinotherapy cycle was able to significantly reduce both ROMs and glucose plasma levels in patients with gastrointestinal disorders who had received a prescription for such a Spa treatment.

Notably, the hydropinotherapy was responsible for ROMs reduction, in turn, associated with an improvement of glycaemic homeostasis.

Oxidative stress plays a fundamental role in the pathogenesis of several chronic disorders and their complications [37]. Overproduction of ROS is associated with glucose homeostasis deficiency, and several molecular mechanisms have been proposed to explain such a link that favours hyperglycaemia and insulin resistance [4,6,40]. Hyperglycaemia itself impairs the capacity of the pancreatic islet cells to secrete insulin, initiating a vicious circle in which the increase in insulin resistance causes a further increase in blood glucose levels [41].

Several studies have demonstrated that hydropinotherapy with sulphurous mineral water is effective to contrast metabolic disorders, especially T2D [19,42] and gastrointestinal diseases [20,43,44].

These benefits are related to the presence of sulphur (in the form of hydrogen sulphide and sulphate ions), magnesium, and bicarbonate ions [45,46,47,48,49,50]. In particular, the antioxidant and hypoglycaemic effects exerted by sulphurous mineral water are essentially due to H_2_S, a recognised precious compound with a great potential for therapeutic applications [51] also owing to its cell signalling function [52,53]. Moreover, in the literature, a considerable amount of evidence is available on the protective role of magnesium salts against oxidative damage by enhancing the efficiency of the antioxidant system represented by the enzymes catalase, glutathione peroxidase, and superoxide dismutase [44,45,48]. It has been shown that magnesium deficiency favours the formation of free radicals and the increase in sympathetic tone thus hindering the activity of insulin. Therefore, a preventive intake of magnesium can avoid the onset of complications in patients with metabolic disorders [54]. Additionally, bicarbonate and calcium ions contained in sulphureous mineral water concur to improve metabolism and motor-secretory activity of the gastrointestinal system preventing ageing [49,55,56].

In our study, another important finding concerns the QoL of the patients who underwent hydropinotherapy. This is important considering that metabolic diseases, especially T2D, impact the patients’ QoL with social and economic negative implications [57]. 

Spa treatments, such as mud-bath therapy or balneotherapy, have been recognised as helpful therapeutic approaches to improve the QoL of patients belonging to several clinical settings [21,35]. 

Contrary to other Spa treatments, data on the effects of hydropinotherapy on QoL are scarce. In our study, we found that a 2-week sulphurous hydropinotherapy was able to significantly improve the QoL perception, measured by the SF-36, both with respect to the levels at baseline and to the control group. In particular, the best effects were found in the improvement of vitality, emotional role functioning, and mental health items. This finding supports the hypothesis that a better glycaemic and antioxidant status could also induce an improvement in QoL.

This study presents some limitations and strengths. First of all, the choice of hydropinotherapy prescription was made by different physicians. This could represent both a limitation, because of the heterogeneity of treatment for similar conditions, but also strength. In fact, in our analysis, we did not find any difference in the prevalence of diseases and drugs administered between the S-HT and No S-HT groups. Another limitation can be the possible beneficial placebo effect in the S-HT group, related to the attendance of the Spa. Indeed, the S-HT group attended the spa only for hydropinotherapy and, therefore, for a really short period. Another limitation is represented by the small sample size of each group, whereas in a similar study, the recruited population has a similar or smaller size. Nonetheless, further studies are necessary to confirm our findings.

The strength is the design of the study that investigated the hydropinotherapy effects on glycaemia and oxidative stress, and also on the QoL in subjects affected by metabolic and/or gastrointestinal disorders. 

## 5. Conclusions

The assessment of the patients’ well-being is now considered a fundamental indicator for monitoring the results of care but also to establish the effectiveness of treatments. Our data suggest that hydropinotherapy could be considered a useful complementary therapeutic approach to improve the glycaemic and oxidant status and also the perceived QoL of patients with important chronic diseases. 

## Figures and Tables

**Table 1:** Main characteristics of the study population.

	S-HT Group *n* = 90	No S-HT Group *n* = 35	*p*
Age, years mean ± SD median (range)	58.1 ± 10.9 57 [23–84]	61.3 ± 10.7 61 [22–84]	0.134
Height, m mean ± SD (range)	1.69 ± 0.08 (1.5–1.9)	1.68 ± 0.07 (1.53–1.89)	0.557
Weight, Kg mean ± SD (range)	75.9 ± 13.6 (50–118.5)	75.5 ± 13.8 (53–124)	0.874
BMI, Kg/m^2^ mean ± SD (range)	26.5 ± 3.6 (17.5–37.2)	26.7 ± 4.3 (17.5–36.9)	0.763
SEX, *n* (%) Male Female	66 (73) 24 (27)	23 (66) 12 (34)	0.398
Chronic gastritis/gastroduodenitis, *n* (%)	38 (42.22)	16 (45.71)	0.723
Gastroesophageal reflux, *n* (%)	25 (27.78)	11 (31.43)	0.686
Hiatal hernia, *n* (%)	27 (30.00)	8 (22.85)	0.425
Number of drugs			
mean ± SD	0.93 ± 1.15	1.22 ± 1.06	0.193
(range)	(0–5)	(0–4)	

**Table 2:** General characteristics of the subpopulation considered for the analysis.

	S-HT Group *n* = 60	No S-HT Group *n* = 35	*p*
Age, years mean ± SD median (range)	59.1 ± 11.2 58 [23–83]	61.3 ± 10.7 61 [22–84]	0.351
Height, m mean ± SD (range)	1.67 ± 0.07 (1.5–1.89)	1.68 ± 0.07 (1.53–1.89)	0.782
Weight, Kg mean ± SD (range)	73.5 ± 12.3 (50–118.5)	75.5 ± 13.8 (53–124)	0.469
BMI, Kg/m^2^ mean ± SD (range)	26.1 ± 3.5 (19.3–37.2)	26.7 ± 4.3 (17.5–36.9)	0.451
Glycaemia at baseline, g/dL			
mean ± SD	125.97 ± 26.92	125.71 ± 27.71	0.965
(range)	(76–198)	(78–177)	
ROMs at baseline, U.Carr.			
mean ± SD	331.25 ± 47.12	331.31 ± 66.15	0.996
(range)	(256–487)	(243–463)	
Sex, *n* (%) Male Female	41 (68.3) 19 (31.7)	23 (66) 12 (34)	0.398
Chronic gastritis/gastroduodenitis, *n* (%)	27 (45.00)	16 (45.71)	0.946
Gastroesophageal reflux, *n* (%)	16 (26.67)	11 (31.43)	0.620
Hyatal hernia, *n* (%)	17 (28.33)	8 (22.86)	0.559
Number of drugs			
mean ± SD	1.08 ± 1.25	1.22 ± 1.06	0.566
(range)	(0–5)	(0–4)	

**Table 3:** Comparison of the scores expressed as mean values ± SD, calculated on the scales of the SF-36 questionnaire administered at baseline and at 2 weeks in S-HT and No S-HT groups.

SF-36 Items	S-HT Group			No S-HT Group		
	at Baseline	at 2 Weeks	*p*	at Baseline	at 2 Weeks	*p*
Physical functioning	86.25 ± 20.08	87.17 ± 19.62	0.801	86.28 ± 24.26	82.23 ± 29.41	0.531
Physical role functioning	75.00 ± 38.51	91.25 ± 23.84	0.006	77.86 ± 33.08	86.43 ± 32.28	0.277
Body pain	67.73 ± 28.17	79.90 ± 25.80	0.015	69.14 ± 23.77	74.31 ± 24.39	0.372
General health perceptions	57.73 ± 16.11	63.65 ± 15.84	0.045	49.77 ± 13.37	54.83 ± 11.20	0.091
Vitality	61.83 ± 20.50	68.50 ± 21.36	0.084	58.14 ± 11.19	59.14 ± 13.48	0.737
Social role functioning	73.35 ± 21.99	82.33 ± 20.79	0.023	70.4 ± 16.81	73.37 ± 20.29	0.507
Emotional role functioning	72.68 ± 40.04	88.82 ± 26.60	0.011	84.69 ± 32.73	86.63 ± 32.56	0.804
Mental health	62.60 ± 18.73	71.53 ± 20.10	0.013	56.11 ± 9.18	56.69 ± 10.60	0.810

## Data Availability

Data are contained within the article and Supplementary Materials.

## Supplementary Materials

The following are available online at https://www.mdpi.com/article/10.3390/antiox10111773/s1, Table S1: Chemical composition of mineral water, Table S2: Reliability and validity of SF-36, Figure S1: logistic regression.
