# Supplementary material for: Hydropinotherapy with Sulphurous Mineral Water as Complementary Treatment to Improve Glucose Metabolism, Oxidative Status, and Quality of Life
Source: Antioxidants (Basel). 2021 Nov 5;10(11):1773. doi: 10.3390/antiox10111773 (PMC8614851; doi:10.3390/antiox10111773)
Supplement: Supplementary file 1 [file antioxidants-10-01773-s001.zip › antioxidants-1396396-supplementary.pdf]

Table S1– Chemical composition of sulphurous, carbogaseous,bicarbonate with calcium and magnesium natural mineral water of the Telese spa (Benevento-Italy).

Chemical composition	Concentration
pH	6.08
fixed residue at 180°C (mg/L)	2080
iodide (mg/L)	<0.02
hydrogen sulfide (H_2S) (mg/L)	11.7
sodium (mg/L)	127
potassium (mg/L)	19.8
calcium (mg/dL)	505
magnesium (mg/L)	83.4
lithium (mg/L)	0.70
chloride (mg/L)	194
iron (mg/L)	0.04
bromide (mg/L)	2.9
bicarbonate (mg/L)	1962
sulphate (mg/L)	37.1
silica (mg/L)	17.1
carbon dioxide (mg/L)	1680

Table S2– Reliability and Validity of SF-36

		Internal consistency reliability	Test-retest reliability	Concurrent Validity
		Cronbach's α	*ICC*	
PC	**Physical functioning**	0.837	0.930	0.701
	**Physical role functioning**	0.834	0.737	0.840
	**Body pain**	0.827	0.808	0.729
	**General health perceptions**	0.824	0.876	0.664
MC	**Vitality**	0.816	0.904	0.789
	**Social role functioning**	0.831	0.820	0.744
	**Emotional role functioning**	0.827	0.742	0.750
	**Mental health**	0.821	0.821	0.780

ICC, Intra-class correlation; PC, physical component; MC, mental component

Logistic regression

Number of obs = **95**
LR chi2(**6**) = **21.63**
Prob > chi2 = **0.0014**
Log likelihood = **-51.704141**
Pseudo R2 = **0.1730**

Group_SHT	Coef.	Std. Err.	z	P>|z|	[95% Conf.	Interval]
Age	.021227	.0234887	0.90	0.366	-.0248099	.067264
BMI	.0083429	.064485	0.13	0.897	-.1180453	.1347312
delta_Glycemia_2_0	.0227688	.0197814	1.15	0.250	-.0160021	.0615396
Delta_ROMs	.0369859	.0154356	2.40	0.017	.0067326	.0672392
N_drug	.1643743	.2104597	0.78	0.435	-.2481191	.5768678
Sex						
F	.3619755	.530955	0.68	0.495	-.6786773	1.402628
_cons	-2.058655	2.108171	-0.98	0.329	-6.190594	2.073284

Figure S1

Logistic regression

Number of obs = **95**
LR chi2(**6**) = **21.63**
Prob > chi2 = **0.0014**
Log likelihood = **-51.704141**
Pseudo R2 = **0.1730**

Group_SHT	Odds Ratio	Std. Err.	z	P>|z|	[95% Conf.	Interval]
Age	**1.021454**	**.0239926**	**0.90**	**0.366**	**.9754953**	**1.069578**
BMI	**1.008378**	**.0650252**	**0.13**	**0.897**	**.8886558**	**1.144229**
delta_Glycemia_2_0	**1.02303**	**.020237**	**1.15**	**0.250**	**.9841253**	**1.063473**
Delta_ROMs	**1.037678**	**.0160172**	**2.40**	**0.017**	**1.006755**	**1.069551**
N_drug	**1.178655**	**.2480595**	**0.78**	**0.435**	**.780267**	**1.780453**
Sex						
F	**1.436164**	**.7625384**	**0.68**	**0.495**	**.5072875**	**4.065872**
_cons	**.1276255**	**.2690564**	**-0.98**	**0.329**	**.0020486**	**7.950895**

Note: **_cons** estimates baseline odds.

Fig. S1
